# Óbitos por câncer de cabeça e pescoço segundo escolaridade no Rio de Janeiro, Brasil, 2010-2018

**DOI:** 10.1590/0102-311XPT140023

**Published:** 2025-03-31

**Authors:** Debora Santos da Silva, Mirian Carvalho Souza, Jonas Eduardo Monteiro dos Santos, Lucas Melo Guimaraes, Geraldo Marcelo da Cunha

**Affiliations:** 1 Instituto Nacional de Câncer, Rio de Janeiro, Brasil.; 2 Escola Nacional de Saúde Pública Sergio Arouca, Fundação Oswaldo Cruz, Rio de Janeiro, Brasil.; 3 Fundação Municipal de Saúde, Teresina, Brasil.

**Keywords:** Neoplasias de Cabeça e Pescoço, Disparidades nos Níveis de Saúde, Escolaridade, Indicadores de Morbimortalidade, Neoplasias Bucais, Head and Neck Neoplasms, Health Status Disparities, Educational Status, Indicators of Morbidity and Mortality, Mouth Neoplasms, Neoplasias de Cabeza y Cuello, Disparidades en el Estado de Salud, Escolaridad, Indicadores de Morbimortalidad, Neoplasias de la Boca

## Abstract

O objetivo foi avaliar as diferenças nas taxas de mortalidade por câncer de cabeça e pescoço de acordo com a escolaridade, no Estado do Rio de Janeiro, Brasil, no período entre 2010 e 2018. Trata-se de um estudo ecológico que utilizou dados de câncer de cabeça e pescoço do Sistema de Informações sobre Mortalidade. Foram comparadas as taxas de mortalidade por câncer de cabeça e pescoço em indivíduos maiores de 40 anos, após a imputação da variável escolaridade. Foram gerados 20 bancos de dados imputados e para cada banco de dados imputados um modelo de regressão binomial negativa foi ajustado por idade, sexo, escolaridade, topografia do tumor, regiões geográficas imediatas e ano do óbito. A partir da combinação dos coeficientes dos 20 modelos ajustados, estimaram-se as taxas e as razões de taxas de mortalidade por câncer de cabeça e pescoço. As taxas de mortalidade por câncer de cabeça e pescoço foram mais altas entre homens com mais de 50 anos e menos de 8 anos de estudo, ultrapassando 40 mortes por 100 mil. Diferenças significativas foram observadas no câncer de orofaringe e cavidade oral, com mortalidade quatro vezes entre os menos escolarizados e cinco vezes entre homens em comparação às mulheres. Na região norte do estado, a mortalidade por câncer de cabeça e pescoço foi pelo menos oito vezes maior entre os menos escolarizados. Observou-se um maior risco de mortalidade por câncer de cabeça e pescoço em indivíduos com baixa escolaridade, especialmente em homens acima de 50 anos e aqueles com residência no interior do estado. Esses resultados ressaltam a importância em considerar as desigualdades em saúde e implementar estratégias de prevenção para reduzir o impacto do câncer de cabeça e pescoço em grupos socialmente mais vulneráveis.

## Abstract (en)

This study aimed to evaluate the differences in mortality rates from head and neck cancer according to schooling in the state of Rio de Janeiro, Brazil, from 2010 to 2018. This ecological study used head and neck cancer data from the Brazilian Mortality Information System. Mortality rates from head and neck cancer in individuals over 40 years of age were compared after inputting the schooling variable. A total of 20 databases were generated for each of which a negative binomial regression model was adjusted for age, sex, education, tumor topography, immediate geographic regions, and year of death. Combining the coefficients of the 20 adjusted models estimated the rates and mortality rate ratios for head and neck cancer. Death rates from head and neck cancer were highest in men aged over 50 years with less than eight years of schooling, exceeding 40 deaths per 100,000. Significant differences occurred in oropharyngeal and oral cavity cancer, with a mortality four times in those with less schooling and five times in men than in women. Northern Rio de Janeiro showed a mortality from head and neck cancer at least eight times higher in those with less schooling. A higher risk of mortality from head and neck cancer occurred in individuals with low schooling, especially in men over 50 years of age and those living in the inner state. These results underscore the importance of considering health inequalities and implementing prevention strategies to reduce the impact of head and neck cancer on more socially vulnerable groups.

## Abstract (es)

El objetivo de este estudio fue evaluar las diferencias en las tasas de mortalidad por cáncer de cabeza y cuello según el nivel de estudios en el estado de Río de Janeiro, Brasil, en el período entre 2010 y 2018. Se trata de un estudio ecológico que utilizó datos sobre cáncer de cabeza y cuello del Sistema de Información de Mortalidad. Las tasas de mortalidad por cáncer de cabeza y cuello en individuos mayores de 40 años se compararon después de la imputación de la variable nivel de estudios. Se generaron 20 bases de datos imputadas, y para cada una se ajustó un modelo de regresión binomial negativa por edad, sexo, nivel de estudios, topografía tumoral, regiones geográficas inmediatas y año de muerte. A partir de la combinación de los coeficientes de los 20 modelos ajustados, se estimaron las tasas y razones de las tasas de mortalidad por cáncer de cabeza y cuello. Las tasas de mortalidad por cáncer de cabeza y cuello fueron más altas entre los hombres mayores de 50 años, con menos de 8 años de estudio, superando las 40 muertes por cada 100.000. Se observaron diferencias significativas en el cáncer de orofaringe y cavidad oral, con una mortalidad cuatro veces entre las personas con bajos niveles de estudios y cinco veces entre los hombres en comparación con las mujeres. En la región norte del estado, la mortalidad por cáncer de cabeza y cuello fue al menos ocho veces mayor entre las personas con bajos niveles de estudios. Se observó un mayor riesgo de mortalidad por esta causa en individuos con bajos niveles de estudios, especialmente en hombres mayores de 50 años y en aquellas personas que viven en otras ciudades del estado. Los resultados subrayan la importancia de considerar las desigualdades en salud y de implementar estrategias de prevención para que se reduzca el impacto del cáncer de cabeza y cuello en los grupos socialmente más vulnerables.

## Introdução

O câncer de cabeça e pescoço está entre as dez neoplasias mais incidentes no mundo. Em 2022, foram estimados 758.020 novos casos de câncer de cabeça e pescoço e 379.069 mortes em 2022 ^1^. O câncer de cabeça e pescoço é considerado um grupo de tumores que afeta diversas áreas, como os lábios, a cavidade oral e a faringe, apesar de não haver consenso de sua definição na literatura ^2^. No Brasil, o câncer de cabeça e pescoço ocupa a oitava posição em termos de incidência com uma estimativa de 15.100 casos novos para cada ano do triênio de 2023-2025. Isso corresponde a um risco ajustado pela população mundial de aproximadamente 4,95 casos por 100 mil habitantes ^3^. Em 2022, foram registrados 8.202 óbitos por câncer de cabeça e pescoço no país, o equivalente a 3,02 óbitos para cada 100 mil habitantes ^4^.

A morbimortalidade por câncer de cabeça e pescoço está relacionada com as desigualdades sociais, colocando a doença como um problema de saúde pública, ou seja, as populações mais vulnerabilizadas estão mais expostas a fatores de riscos para a doença como o tabagismo e o consumo de álcool ^5^ ^,^ ^6^. Do mesmo modo, a mortalidade é maior nos grupos sociais que enfrentam maiores barreiras de acesso aos serviços de diagnóstico e tratamento oncológico em tempo oportuno ^6^ ^,^ ^7^.

Estudos em países desenvolvidos mostram que a escolaridade impacta tanto nas chances de exposição aos fatores de risco para o câncer de cabeça e pescoço, quanto na capacidade de lidar com o agravamento da doença já instalada ^2^ ^,^ ^6^. No entanto, a relação entre escolaridade e morbimortalidade por câncer de cabeça e pescoço em países em desenvolvimento como o Brasil, onde a escolaridade é um determinante crucial de emprego e renda ^8^, ainda não é bem esclarecida ^9^ ^,^ ^10^.

Nesse contexto, dados de uma pesquisa nacional revelam que o Rio de Janeiro está entre os estados com maior desigualdade no Brasil, ocupando a 4ª posição em 2023, com um índice de Gini de 0,54 e uma renda *per capita* de R$ 2.367,00 ^11^. O índice de desenvolvimento da educação básica (IDEB) do estado apresentou queda, passando de 3,9 em 2022 para 3,3 em 2023, abaixo da meta de 4,6 para o ensino básico, e o Ensino Médio foi o pior avaliado da Região Sudeste ^12^.

Frente aos elevados índices de desigualdade social presentes no Estado do Rio de Janeiro, o objetivo desse estudo foi verificar a relação entre os níveis de escolaridade e as taxas de mortalidade por câncer de cabeça e pescoço no estado, no período de 2010-2018.

## Método

Trata-se de um estudo ecológico em que foram analisados os óbitos por câncer de cabeça e pescoço entre residentes no Estado do Rio de Janeiro de 2010 a 2018, segundo as 14 regiões geográficas imediatas (RGIs) de residência.

As RGIs consideram um agrupamento mínimo de municípios e são capazes de capturar, nas análises estatísticas, as diferenças socioeconômicas e de acesso ao sistema de saúde dentro do Estado do Rio de Janeiro. Essa nova divisão substituiu as microrregiões estaduais. As 14 RGIs analisadas foram: Rio de Janeiro, Angra do Reis, Rio Bonito, Volta Redonda-Barra Mansa, Resende, Valença, Petrópolis, Nova Friburgo, Três Rios-Paraíba do Sul, Campos dos Goytacazes, Itaperuna, Santo Antônio de Pádua, Cabo Frio e Macaé-Rio das Ostras ^13^.

Os dados sobre os óbitos por neoplasia maligna de cabeça e pescoço foram obtidos nas bases de dados do Sistema de Informações sobre Mortalidade (SIM) do Ministério da Saúde ^14^. Foram considerados elegíveis os óbitos cuja causa básica registrada, conforme a 10ª revisão da Classificação Internacional das Doenças (CID-10) ^15^ era neoplasia de: lábio (C00.0, C00.1); cavidade oral (C00, C02-C06); glândulas salivares (C07-C08); orofaringe (C01, C09-C10); nasofaringe (C11); hipofaringe (C12-C13); ou neoplasias malignas de outros sítios mal definidos do lábio, da cavidade oral e da faringe (C14.0-C14.8).

Para calcular as taxas de mortalidade, os quantitativos populacionais foram obtidos do Censo 2010 (2010) ^16^ e das projeções populacionais (2011-2018) ^17^ do Instituto Brasileiro de Geografia e Estatística (IBGE). O Censo de 2010 ^16^ foi a penúltima contagem oficial de população realizada no Brasil, e suas informações detalham o perfil demográfico e socioeconômico do país. As projeções populacionais subsequentes, elaboradas pelo IBGE, são baseadas em tendências de crescimento populacional e ajudam a estimar a evolução da população brasileira nos anos seguintes, permitindo o cálculo de indicadores de saúde como as taxas de mortalidade.

Na análise de dados, as seguintes características foram avaliadas: sexo (masculino e feminino), faixa etária (40-49, 50-59, 60-69, 70-79, 80 anos ou mais), escolaridade (menos de 8 ou 8 ou mais anos de estudo), raça/cor (branca, não branca), estado civil (solteiro, casado/união consensual, viúvo/separado), topografia do tumor (cabeça e pescoço - C00-C14; cavidade oral - C00, C02-C06; orofaringe - C01, C09-C10; e outros tumores de cabeça e pescoço - C07-C08, C10, C12-C13, C14.0-C14.8), ano do óbito e RGI de residência.

Métodos de imputação múltipla foram elaborados para as variáveis escolaridade, estado civil e raça/cor que apresentaram mal preenchimento no banco de dados do SIM. Ao final do processo, foram gerados 20 bancos imputados pelo método de imputação múltipla por equações encadeadas ^18^. Esta técnica assume, após controlar todas as variáveis incluídas no modelo de imputação, que os dados faltantes se distribuem aleatoriamente. A imputação permite que todos os indivíduos sejam considerados nas análises, aumentando a precisão das estimativas ^19^ ^,^ ^20^ ^,^ ^21^. Em função da logística de imputação dos dados, a raça/cor foi categorizada em dois subgrupos e a escolaridade em dois grupos.

Os quantitativos populacionais foram agregados por sexo, faixa etária e RGI para cada ano de óbito entre 2010 e 2018 ^14^. Cada uma dessas populações nos estratos foi dividida em duas outras, aqueles com menos de 8 anos de estudo e aqueles com 8 ou mais anos de estudo, aplicando-se a proporção descrita a seguir. Devido a oscilações de natureza aleatória nas estimativas das populações entre os anos, modelos lineares foram utilizados para o ajuste das populações ao longo dos anos em cada um dos estratos. Os valores preditos dos modelos foram assumidos como os verdadeiros tamanhos das populações dentro de cada estrato. A partir daí, foram estimadas as proporções populacionais nas categorias da variável escolaridade (menos de 8 e 8 ou mais anos de estudo) por sexo, idade e ano do óbito para o Estado do Rio de Janeiro. A aplicação dessas proporções nas populações projetadas das RGI permitiu calcular as respectivas populações sob risco (pessoas-ano) dentro das categorias de cada um dos estratos das variáveis consideradas na análise.

As taxas e razões de taxas (RT) de mortalidade câncer de cabeça e pescoço foram calculadas considerando a distribuição dos óbitos e a população sob risco dentro das combinações dos estratos de RGI, ano do óbito, sexo, idade e escolaridade, após o processo de imputação das variáveis com dados faltantes.

Modelos de regressão binomial negativa foram ajustados para cada banco contendo os dados agregados nos estratos, tendo o *log* do total de pessoas-ano como *offset* nos modelos. O modelo binomial negativo foi utilizado pelo fato da distribuição dos óbitos por câncer de cabeça e pescoço apresentarem elevada variância. A combinação (*pooling*) das estimativas dos coeficientes dos 20 modelos ajustados permitiu estimar as taxas e as RTs de mortalidade por câncer de cabeça e pescoço segundo as categorias de escolaridade com seus respectivos intervalos de 95% de confiança (IC95%) ^21^.

Para a imputação foram utilizados os pacotes *mice* (*multivariate imputation by chained equations*) ^18^ e *broom.mixed* (*tidying methods for mixed models*) ^22^, do software R 4.4.1 (http://www.r-project.org). Para a construção dos mapas, foram utilizados os pacotes *tmap* (*thematic map in R*) ^23^ e *sf* (*simple features for R*) ^24^. O estudo utilizou dados de vigilância em saúde e estes foram analisados de forma anônima e estão disponíveis de forma irrestrita.

## Resultados

Entre 2010 e 2018, no Estado do Rio de Janeiro, ocorreram 6.429 mortes por câncer de cabeça e pescoço em indivíduos com 40 anos ou mais, o equivalente a 97,3% do total de óbitos considerando todas as idades. Durante o período de análise, a taxa anual média de mortalidade bruta foi de 12,8 por 100 mil habitantes. Contudo, essa taxa foi quase quatro vezes maior entre homens (20,1 por 100 mil) em comparação com mulheres (5,5 por 100 mil). Ademais, a taxa bruta anual de mortalidade entre aqueles com menor nível de escolaridade (21,7 por 100 mil) foi quatro vezes superior à dos mais escolarizados (4,0 por 100 mil). As topografias mais frequentes foram o câncer de cavidade oral (39,6%) e orofaringe (36,6%). Houve predomínio de óbitos em pessoas classificadas como brancas (53,3%), casadas (40,5%) e residentes na RGI Rio de Janeiro (75,3%) (Tabela 1).

**Tabela 1:** Número e proporção dos óbitos por câncer de cabeça e pescoço estratificado por escolaridade, segundo sexo, faixa etária, estado civil, raça/cor, topografia do tumor e regiões de influência *. Estado do Rio de Janeiro, Brasil, 2010-2018.

Variáveis	Total [n (%)]	Escolaridade (anos de estudo) [n (%)]		
		SI	Menos de 8	8 ou mais
Total	6.429 (100,0)	625 (9,7)	4.357 (67,8)	1.447 (22,5)
Sexo				
Masculino	5.076 (79,0)	508 (81,3)	3.442 (79,0)	1.126 (77,8)
Feminino	1.353 (21,0)	117 (18,7)	915 (21,0)	321 (22,2)
Faixa etária (anos)				
40-49	586 (9,1)	61 (9,8)	414 (9,5)	111 (7,7)
50-59	1.926 (30,0)	165 (26,4)	1.324 (30,4)	437 (30,2)
60-69	1.996 (31,0)	198 (31,7)	1.325 (30,4)	473 (32,7)
70-79	1.148 (17,9)	110 (17,6)	775 (17,8)	263 (18,2)
80 ou mais	773 (12,0)	91 (14,6)	519 (11,9)	163 (11,3)
Estado civil				
Sem informação	43 (0,7)	16 (2,6)	22 (0,5)	5 (0,3)
Solteiro	2.104 (32,7)	198 (31,7)	1.564 (35,9)	342 (23,6)
Casado/União consensual	2.603 (40,5)	224 (35,8)	1.683 (38,6)	696 (48,1)
Viúvo/Separado	1.613 (25,1)	150 (24,0)	1.066 (24,5)	397 (27,4)
Ignorado	66 (1,0)	37 (5,9)	22 (0,5)	7 (0,5)
Raça/Cor				
Sem informação	63 (1,0)	10 (1,6)	37 (0,8)	16 (1,1)
Branca	3.426 (53,3)	331 (53,0)	2.102 (48,4)	993 (68,6)
Preta	956 (14,9)	104 (16,6)	728 (16,7)	124 (8,6)
style="text-indent:12px" Amarela/Indígena	9 (0,1)	2 (0,3)	2 (0,0)	5 (0,3)
Parda	1.975 (30,7)	178 (28,5)	1.488 (34,2)	309 (21,4)
Topografia do tumor				
Glândulas salivares	332 (5,2)	30 (4,8)	187 (4,3)	115 (7,9)
Cavidade oral	2.546 (39,6)	260 (41,6)	1.748 (40,1)	538 (37,2)
Hipofaringe	354 (5,5)	27 (4,3)	254 (5,8)	73 (5,0)
Lábio	7 (0,1)	0 (0,0)	7 (0,2)	0 (0,0)
Nasofaringe	263 (4,1)	16 (2,6)	149 (3,4)	98 (6,8)
Orofaringe	2.356 (36,6)	206 (33,0)	1.644 (37,7)	506 (35,0)
Outras localizações mal definidas **	571 (8,9)	86 (13,8)	368 (8,4)	117 (8,1)
Regiões de Influência (RGI)				
Angra dos Reis	61 (0,9)	3 (0,5)	51 (1,2)	7 (0,5)
Cabo Frio	167 (2,6)	18 (2,9)	116 (2,7)	33 (2,3)
Campos dos Goytacazes	203 (3,2)	31 (5,0)	154 (3,5)	18 (1,2)
Itaperuna	96 (1,5)	7 (1,1)	76 (1,7)	13 (0,9)
Macaé/Rio das Ostras	127 (2,0)	18 (2,9)	90 (2,1)	19 (1,3)
Nova Friburgo	121 (1,9)	58 (9,3)	55 (1,3)	8 (0,6)
Petrópolis	236 (3,7)	54 (8,6)	142 (3,3)	40 (2,8)
Resende	76 (1,2)	9 (1,4)	53 (1,2)	14 (1,0)
Rio Bonito	53 (0,8)	4 (0,6)	43 (1,0)	6 (0,4)
Rio de Janeiro	4.838 (75,3)	348 (55,7)	3.280 (75,3)	1.210 (83,6)
Santo Antônio de Pádua	44 (0,7)	9 (1,4)	26 (0,6)	9 (0,6)
Três Rios/Paraíba do Sul	58 (0,9)	30 (4,8)	25 (0,6)	3 (0,2)
Valença	75 (1,2)	10 (1,6)	55 (1,3)	10 (0,7)
Volta Redonda/Barra Mansa	274 (4,3)	26 (4,2)	191 (4,4)	57 (3,9)

RGI: regiões geográficas imediatas; SI: sem informação.

* Dados não imputados;

** Neoplasias malignas de outros sítios mal definidos do lábio, cavidade oral e faringe.

Em relação a escolaridade, aproximadamente 7 em cada 10 óbitos por câncer de cabeça e pescoço ocorreram entre pessoas com menos de 8 anos de estudo. Relativamente aos mais escolarizados, os menos escolarizados apresentaram maior percentual de mortes entre solteiros, pretos e pardos. Comparado a população geral, aqueles sem informação sobre a escolaridade tiveram maior percentual de mortes entre aqueles sem informação sobre estado civil e com a localização do tumor mal definida. Para cerca de 50% dos óbitos entre residentes das RGIs Três Rios-Paraíba do Sul e Nova Friburgo, a escolaridade não foi registrada (Tabela 1).

Após a realização da imputação dos dados, foram estimadas taxas de mortalidade por câncer de cabeça e pescoço para ambos os sexos de 23,88 óbitos por 100 mil (IC95%: 22,05-25,86) entre os menos escolarizados e 4,62 óbitos por 100 mil (IC95%: 4,12-5,19) entre os mais escolarizados. Entre homens com baixa escolaridade e faixas de idade acima de 50 anos, as taxas de mortalidade estiveram acima de 40 óbitos por 100 mil e diminuíram a partir dos 69 anos. Esse padrão é observado quando as taxas foram estratificadas por topografia (Tabela 2).

**Tabela 2:** Taxas por 100 mil pessoas-ano e razões de taxas (RT) brutas de mortalidade por de câncer cabeça e pescoço, preditas por imputação *, estratificadas por escolaridade, segundo topografia do tumor, sexo e faixa etária. Estado do Rio de Janeiro, Brasil, 2010-2018.

Topografia do tumor (CID-10)/Sexo	Faixa etária (anos)	Escolaridade [taxa (IC95%)]		RT
		Menos de 8 anos	8 anos ou mais	
Cabeça e pescoço (C00-C14)				
Ambos	40-49	9,35 (7,82-11,19)	0,78 (0,58-1,05)	11,94 (9,82-14,66)
	50-59	24,68 (21,46-28,39)	3,50 (2,87-4,27)	9,38 (8,42-10,47)
	60-69	28,19 (24,48-32,46)	6,75 (5,53-8,22)	8,95 (8,06-9,37)
	70-79	27,45 (23,46-32,13)	9,00 (7,08-11,45)	3,96 (3,47-4,54)
	80 ou mais	33,62 (28,24-40,01)	12,50 (9,42-16,59)	12,39 (10,39-14,88)
	Subtotal	23,88 (22,05-25,86)	4,62 (4,12-5,19)	5,16 (4,89-5,45)
Masculino	40-49	13,91 (12,43-15,56)	1,14 (0,90-1,45)	12,21 (10,37-14,45)
	50-59	43,75 (40,59-47,16)	7,00 (6,23-7,88)	8,31 (7,69-8,99)
	60-69	52,22 (48,40-56,35)	14,49 (12,97-16,19)	7,73 (7,18-8,32)
	70-79	46,90 (42,69-51,52)	17,61 (15,20-20,40)	3,58 (3,26-3,95)
	80 ou mais	42,89 (37,66-48,84)	24,91 (20,19-30,73)	7,93 (6,96-9,08)
	Subtotal	39,78 (36,74-43,07)	7,78 (6,94-8,72)	5,11 (4,90-5,33)
Feminino	40-49	2,09 (1,62-2,69)	0,54 (0,39-0,73)	3,88 (2,94-5,15)
	50-59	5,46 (4,68-6,37)	1,39 (1,11-1,73)	5,20 (4,33-6,28)
	60-69	7,36 (6,41-8,45)	2,22 (1,75-2,82)	7,11 (5,89-8,62)
	70-79	8,79 (7,58-10,20)	4,20 (3,23-5,47)	1,87 (1,52-2,32)
	80 ou mais	20,67 (18,21-23,46)	10,37 (8,14-13,22)	9,16 (7,51-11,26)
	Subtotal	8,45 (7,45-9,58)	1,92 (1,59-2,31)	4,39 (4,03-4,79)
Cavidade oral (C00, C02-C06)				
Ambos	40-49	3,31 (2,62-4,18)	0,32 (0,22-0,46)	10,43 (7,66-14,50)
	50-59	9,95 (8,43-11,76)	1,21 (0,92-1,58)	10,89 (9,11-13,12)
	60-69	10,45 (8,80-12,41)	3,16 (2,48-4,01)	7,08 (6,05-8,32)
	70-79	10,37 (8,54-12,60)	3,66 (2,71-4,93)	3,93 (3,20-4,88)
	80 ou mais	15,29 (12,43-18,81)	6,03 (4,31-8,43)	11,68 (9,07-15,26)
	Subtotal	9,34 (8,48-10,28)	1,88 (1,63-2,17)	4,96 (4,56-5,41)
Masculino	40-49	4,86 (4,08-5,78)	0,49 (0,34-0,71)	9,87 (7,68-12,84)
	50-59	17,54 (15,85-19,40)	2,39 (1,99-2,88)	9,74 (8,55-11,12)
	60-69	19,51 (17,54-21,70)	5,44 (4,60-6,45)	7,68 (6,82-8,67)
	70-79	17,51 (15,27-20,08)	5,86 (4,58-7,50)	4,33 (3,68-5,13)
	80 ou mais	16,54 (13,62-20,09)	9,24 (6,64-12,86)	8,23 (6,65-10,27)
	Subtotal	14,98 (13,54-16,57)	2,90 (2,49-3,37)	5,16 (4,82-5,53)
Feminino	40-49	0,83 (0,56-1,23)	0,17 (0,10-0,29)	4,87 (3,04-8,02)
	50-59	2,05 (1,62-2,60)	0,43 (0,29-0,63)	6,34 (4,61-8,87)
	60-69	3,14 (2,59-3,82)	1,30 (0,96-1,77)	5,15 (3,98-6,70)
	70-79	4,38 (3,61-5,32)	2,01 (1,40-2,89)	1,46 (1,07-2,02)
	80 ou mais	12,52 (10,82-14,49)	5,55 (4,02-7,65)	10,30 (7,90-13,64)
	Subtotal	4,14 (3,52-4,85)	0,97 (0,77-1,22)	4,26 (3,78-4,81)
Orofaringe (C01, C09-C10)				
Ambos	40-49	3,96 (3,10-5,04)	0,25 (0,16-0,40)	15,81 (11,33-22,78)
	50-59	9,39 (7,78-11,33)	1,51 (1,15-1,99)	8,24 (6,99-9,78)
	60-69	10,85 (9,00-13,08)	2,38 (1,79-3,16)	9,79 (8,22-11,73)
	70-79	9,76 (7,86-12,13)	2,91 (2,06-4,11)	4,63 (3,69-5,89)
	80 ou mais	7,76 (5,84-10,26)	4,07 (2,68-6,16)	8,67 (6,32-12,12)
	Subtotal	8,36 (7,54-9,28)	1,61 (1,38-1,88)	5,18 (4,73-5,69)
Masculino	40-49	6,06 (5,18-7,10)	0,37 (0,25-0,56)	16,28 (12,38-21,85)
	50-59	17,57 (15,84-19,50)	2,80 (2,34-3,35)	8,33 (7,37-9,43)
	60-69	21,38 (19,27-23,73)	5,80 (4,92-6,84)	7,90 (7,04-8,88)
	70-79	18,47 (16,14-21,14)	5,83 (4,57-7,44)	4,33 (3,68-5,13)
	80 ou mais	12,06 (9,61-15,12)	7,62 (5,31-10,95)	7,30 (5,75-9,36)
	Subtotal	15,30 (13,83-16,94)	2,90 (2,49-3,37)	5,27 (4,93-5,65)
Feminino	40-49	0,64 (0,41-0,99)	0,14 (0,08-0,26)	4,52 (2,68-7,85)
	50-59	2,04 (1,61-2,58)	0,47 (0,32-0,67)	5,77 (4,23-7,97)
	60-69	2,44 (1,96-3,04)	0,65 (0,42-1,01)	8,01 (5,72-11,46)
	70-79	1,86 (1,38-2,51)	0,92 (0,54-1,58)	3,21 (2,13-5,05)
	80 ou mais	3,69 (2,81-4,83)	2,55 (1,58-4,14)	6,72 (4,44-10,46)
	Subtotal	2,02 (1,73-2,35)	0,51 (0,40-0,64)	3,95 (3,35-4,69)
Outros tumores de cabeça e pescoço (C07-C08, C10, C12-C13, C14.0-C14.8) **				
Ambos	40-49	1,45 (1,05-2,00)	0,19 (0,12-0,31)	7,65 (5,07-11,93)
	50-59	4,36 (3,47-5,48)	0,89 (0,65-1,22)	6,51 (5,21-8,19)
	60-69	5,22 (4,17-6,54)	1,44 (1,04-1,99)	7,75 (6,16-9,83)
	70-79	4,89 (3,75-6,38)	2,21 (1,51-3,23)	2,86 (2,18-3,82)
	80 ou mais	5,91 (4,38-7,97)	2,18 (1,30-3,67)	12,46 (8,26-19,58)
	Subtotal	4,17 (3,68-4,73)	1,00 (0,83-1,19)	4,16 (3,70-4,69)
Masculino	40-49	2,31 (1,82-2,94)	0,23 (0,14-0,39)	10,03 (6,98-14,83)
	50-59	7,72 (6,73-8,85)	1,56 (1,25-1,95)	6,59 (5,57-7,83)
	60-69	9,97 (8,71-11,42)	2,91 (2,32-3,64)	7,33 (6,23-8,67)
	70-79	9,20 (7,70-10,99)	4,40 (3,35-5,79)	2,54 (2,09-3,11)
	80 ou mais	9,99 (7,84-12,75)	3,78 (2,24-6,38)	12,25 (8,92-17,22)
	Subtotal	7,37 (6,49-8,37)	1,75 (1,47-2,10)	4,21 (3,85-4,61)
Feminino	40-49	0,46 (0,27-0,79)	0,16 (0,09-0,28)	2,85 (1,64-4,96)
	50-59	0,96 (0,68-1,35)	0,35 (0,23-0,53)	3,63 (2,45-5,44)
	60-69	1,27 (0,93-1,72)	0,19 (0,08 -0,44)	14,51 (8,18-28,22)
	70-79	1,53 (1,10-2,14)	0,78 (0,42-1,44)	1,79 (1,11-3,02)
	80 ou mais	2,04 (1,42-2,93)	1,09 (0,53-2,23)	8,88 (4,83-17,67)
	Subtotal	1,17 (0,98-1,41)	0,31 (0,24-0,41)	3,78 (3,05-4,71)

CID-10: Classificação Internacional de Doenças, 10ª revisão; IC95%: intervalo de 95% de confiança.

* Realizada a imputação múltipla da variável escolaridade;

** Glândulas salivares, hipofaringe, lábio, nasofaringe, outras localizações mal definidas do lábio, cavidade oral e faringe.

Observou-se um decréscimo nas RTs em relação aos anos de escolaridade à medida que a idade aumenta até os 79 anos, tanto para homens quanto para mulheres. No entanto, a partir de 80 anos ou mais de idade, as RTs voltam a aumentar, com exceção das taxas de mulheres com tumores de orofaringe, que apresentam um aumento nas RTs até os 69 anos, seguido por um declínio no grupo de idade 70-79 anos e, novamente, um aumento nas mulheres de 80 anos ou mais (Tabela 2).

Ao considerar a estratificação por sexo e tipo de tumor, a maior diferença nas taxas entre mais e menos escolarizados de ambos os sexos foi observada entre aqueles que vieram a óbito por câncer de orofaringe (RT = 5,18; IC95%: 4,73-5,69), seguido por câncer de cavidade oral (RT = 4,96; IC95%: 4,56-5,41). No grupo das mulheres, as maiores razões de taxas de mortalidade entre menos e mais escolarizados foram observadas para os tumores de cavidade oral (RT = 4,26; IC95%: 3,78-4,81) (Tabela 2).

Ao considerar os modelos brutos, estimados após a imputação dos dados, os óbitos ocorridos entre indivíduos menos escolarizados, sexo masculino, e o avanço da idade mostraram associação com taxas aumentadas de mortalidade por câncer de cabeça e pescoço. Embora o ano do óbito não tenha se mostrado estatisticamente significativo no modelo bruto, ele foi mantido no modelo ajustado (Tabela 3).

**Tabela 3:** Modelo bruto de regressão binomial negativa da mortalidade estimada para câncer de cabeça e pescoço, com imputação da variável escolaridade, para o total e por sexo. Estado do Rio de Janeiro, Brasil, 2010-2018.

Variáveis	Total		Homens		Mulheres	
	RT	IC95%	RT	IC95%	RT	IC95%
Escolaridade (anos de estudo)						
8 ou mais *	1,00		1,00		1,00	
Menos de 8	5,16	4,48-5,96 **	5,11	4,43-5,90 **	4,41	3,50-5,54 **
Idade (anos)						
40-50 *	1,00		1,00		1,00	
50-59	3,15	2,55-3,88 **	3,24	2,60-4,04 **	2,72	2,02-3,67 **
60-69	4,16	3,37-5,14 **	4,27	3,42-5,33 **	4,30	3,19-5,79 **
70-79	4,59	3,67-5,73 **	4,39	3,47-5,57 **	6,68	4,93-9,04 **
80 ou mais	5,83	4,60-7,39 **	4,68	3,58-6,12 **	15,89	11,83-21,36 **
Ano do óbito						
2010 *	1,00		1,00		1,00	
2011	1,15	0,85-1,57	1,05	0,74-1,48	1,52	0,93-2,48
2012	1,03	0,75-1,40	0,98	0,69-1,38	1,35	0,82-2,21
2013	1,05	0,77-1,43	1,02	0,72-1,44	1,29	0,79-2,12
2014	0,99	0,72-1,35	0,94	0,67-1,33	1,16	0,70-1,91
2015	1,17	0,86-1,58	1,11	0,79-1,56	1,44	0,89-2,34
2016	1,06	0,78-1,44	1,06	0,76-1,49	1,04	0,63-1,72
2017	1,11	0,81-1,50	1,09	0,78-1,53	1,14	0,69-1,87
2018	0,99	0,73-1,35	0,92	0,66-1,30	1,34	0,83-2,18
Região de influência (RGI)						
Rio de Janeiro *	1,00		1,00		1,00	
Angra dos Reis	0,69	0,47-1,00	0,71	0,47-1,07	0,46	0,22-0,94 **
Cabo Frio	0,71	0,52-0,99 **	0,76	0,54-1,08	0,49	0,30-0,81 **
Campos dos Goytacazes	0,75	0,55-1,02	0,75	0,53-1,06	0,7	0,46-1,08
Itaperuna	0,98	0,70-1,38	1,05	0,72-1,53	0,68	0,38-1,22
Macaé/Rio da Ostras	0,79	0,57-1,11	0,71	0,49-1,03	0,96	0,61-1,52
Nova Friburgo	0,70	0,50-0,97 **	0,76	0,53-1,09	0,44	0,25-0,78 **
Petrópolis	0,96	0,70-1,30	0,97	0,69-1,36	0,89	0,59-1,36
Resende	0,92	0,64-1,31	0,82	0,55-1,23	1,21	0,72-2,03
Rio Bonito	0,81	0,55-1,20	0,8	0,52-1,23	0,78	0,40-1,52
Santo Antônio de Pádua	0,62	0,41-0,93	0,62	0,39-0,98 **	0,62	0,30-1,27
Três Rios/Paraíba do Sul	0,77	0,53-1,12	0,78	0,51-1,18	0,77	0,41-1,44
Valença	0,83	0,58-1,18	0,86	0,58-1,28	0,77	0,43-1,38
Volta Redonda/Barra Mansa	0,92	0,67-1,24	0,95	0,68-1,33	0,74	0,49-1,12

IC95%: intervalo de 95% de confiança; RGI: regiões geográficas imediatas; RT: razão de taxas de mortalidade por câncer de cabeça e pescoço.

* Categorias de referência;

** Valor de p < 0,05.

Na análise ajustada considerando escolaridade, faixa etária, ano do óbito e região de influência, a taxa de mortalidade estimada entre indivíduos menos escolarizados foi quatro vezes a dos mais escolarizados (RT = 4,10; IC95%: 3,73-4,51). Além disso, a taxa de mortalidade entre indivíduos com 80 anos ou mais foi mais de sete vezes a taxa em indivíduos com idade entre 40 e 49 anos (RT = 7,49; IC95%: 6,43-8,73). Houve também uma diferença significativa entre sexos, no qual a taxa de mortalidade entre homens foi cinco vezes a taxa entre mulheres (RT = 5,07; IC95%: 4,63-5,55) (Tabela 4).

**Tabela 4:** Modelo ajustado * de regressão binomial negativa da mortalidade estimada para câncer de cabeça e pescoço, com imputação da variável escolaridade, para o total e por sexo. Estado do Rio de Janeiro, Brasil, 2010-2018.

Variáveis	Total		Homens		Mulheres	
	RT	IC95%	RT	IC95%	RT	IC95%
Escolaridade (anos de estudo)						
8 ou mais **	1,00		1,00		1,00	
Menos de 8	4,10	3,73-4,51 ***	4,47	4,03-4,97 ***	2,86	2.49-3,28 ***
Idade (anos)						
40-50 **	1,00		1,00		1,00	
50-59	3,23	2,82-3,71 ***	3,57	3,07-4,15 ***	2,67	2,12-3,36 ***
60-69	4,53	3,94-5,20 ***	5,02	4,31-5,84 ***	3,89	3,10-4,89 ***
70-79	5,00	4,32-5,78 ***	4,98	4,23-5,85 ***	5,20	4,11-6,57 ***
80 ou mais	7,49	6,43-8,73	5,18	4,31-6,22 ***	12,22	9,73-15,33 ***
Ano do óbito						
2010 **	1,00		1,00		1,00	
2011	1,10	0,92-1,31	1,01	0,83-1,22	1,37	1,06-1,76 ***
2012	1,02	0,86-1,22	0,92	0,76-1,12	1,33	1,03-1,71 ***
2013	1,06	0,89-1,26	0,94	0,77-1,14	1,35	1,05-1,73 ***
2014	0,96	0,80-1,14	0,91	0,75-1,10	1,13	0,87-1,46
2015	1,11	0,93-1,31	1,01	0,84-1,23	1,32	1,03-1,70 ***
2016	1,00	0,84-1,18	0,96	0,79-1,17	1,07	0,82-1,39
2017	0,98	0,82-1,17	0,93	0,76-1,12	1,01	0,78-1,32
2018	0,97	0,82-1,15	0,84	0,69-1,02	1,28	1,00-1,65
Região de influência (RGI)						
Rio de Janeiro **	1,00		1,00		1,00	
Angra dos Reis	0,81	0,62-1,06	0,81	0,61-1,09	0,77	0,40-1,48
Cabo Frio	0,81	0,68-0,97 ***	0,84	0,69-1,02	0,72	0,49-1,06
Campos dos Goytacazes	0,80	0,68-0,94 ***	0,78	0,65-0,94 ***	0,90	0,66-1,23
Itaperuna	1,07	0,86-1,34	1,12	0,88-1,43	0,93	0,56-1,55
Macaé/Rio da Ostras	0,89	0,73-1,09	0,79	0,63-0,99 ***	1,34	0,95-1,91
Nova Friburgo	0,78	0,63-0,95 ***	0,83	0,67-1,03	0,60	0,37-0,96 ***
Petrópolis	1,08	0,93-1,27	1,09	0,91-1,30	1,10	0,82-1,47
Resende	1,08	0,85-1,38	0,93	0,70-1,24	1,71	1,12-2,61 ***
Rio Bonito	0,95	0,71-1,26	0,91	0,66-1,25	1,16	0,64-2,11
Santo Antônio de Pádua	0,76	0,56-1,03	0,74	0,52-1,05	0,85	0,44-1,64
Três Rios/Paraíba do Sul	0,93	0,70-1,22	0,89	0,65-1,21	1,08	0,63-1,88
Valença	0,95	0,75-1,22	0,94	0,71-1,24	1,04	0,64-1,71
Volta Redonda/Barra Mansa	0,97	0,84-1,13	1,00	0,85-1,18	0,92	0,69-1,23

IC95%: intervalo de 95% de confiança; RGI: regiões geográficas imediatas; RT: razão de taxas de mortalidade por câncer de cabeça e pescoço.

* Realizado o ajuste do modelo por sexo, idade, escolaridade, ano do óbito e RGI;

** Categorias de referência;

*** Valor de p < 0,05.

Ao ajustar as RTs separadamente por sexo, homens menos escolarizados apresentaram taxa de mortalidade de 4,5 vezes a taxa dos mais escolarizados (RT = 4,47; IC95%: 4,03-4,97). Já entre mulheres menos escolarizadas, a taxa de mortalidade foi aproximadamente três vezes a taxa das mais escolarizadas (RT = 2,86; IC95%: 2,49-3,28). As taxas de mortalidade entre homens acima de 60 anos não apresentaram variação significativa com o aumento da idade, apesar de serem relativamente mais altas. Por outro lado, entre mulheres, as taxas de mortalidade aumentaram gradualmente à medida que a idade avançou. Homens com 80 anos ou mais apresentaram uma taxa ajustada de mortalidade 5,18 vezes a dos mais jovens (RT = 5,18; IC95%: 4,31-6,22). Já para mulheres na mesma faixa etária, a taxa de mortalidade por câncer de cabeça e pescoço foi 12,2 vezes a taxa das mais jovens (RT = 12,22; IC95%: 9,73-15,33) (Tabela 4).

**Figura 1:**
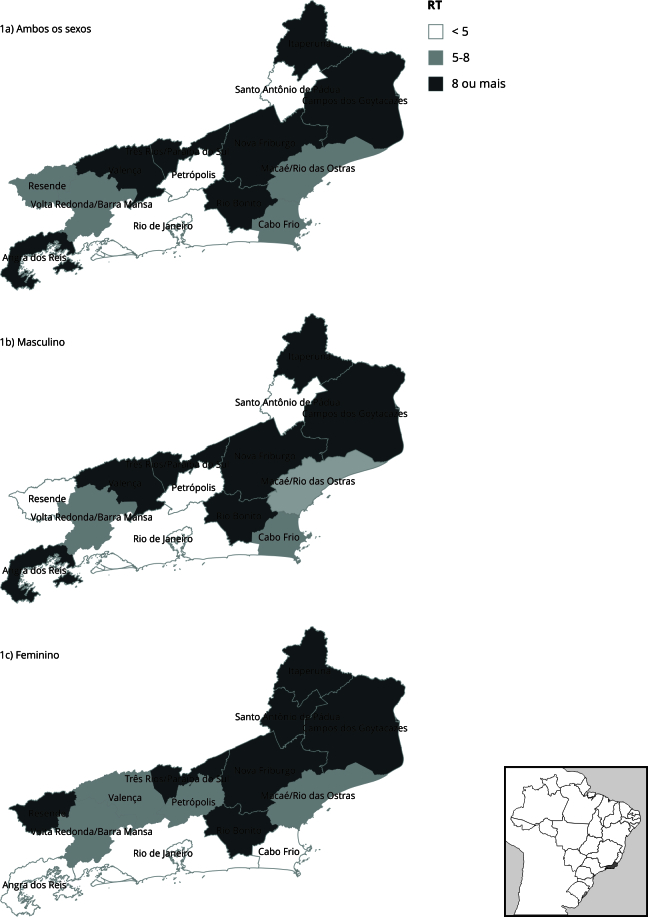
Distribuição espacial das razões de taxas (RT) de mortalidade por câncer de cabeça e pescoço segundo escolaridade, preditas por imputação para as regiões de influência. Estado do Rio de Janeiro, Brasil, 2010-2018.

Nas RGIs localizadas ao norte do estado independente do sexo, as taxas de mortalidade por câncer de cabeça e pescoço entre os menos escolarizados foi pelo menos oito vezes a taxa dos mais escolarizados. Apesar da RGI Rio de Janeiro concentrar dois terços dos óbitos por esse grupo de neoplasias, essa é uma das regiões que apresenta menor diferencial nas taxas de mortalidade entre mais e menos escolarizados (Figura 1).

## Discussão

Neste estudo, verificou-se que a maioria das mortes relacionadas ao câncer de cabeça e pescoço ocorreram em indivíduos com menos de 8 anos de escolaridade, independentemente das demais variáveis analisadas. Notavelmente, foram observadas taxas mais elevadas em homens acima de 50 anos, com menos de 8 anos de estudo. A cada três óbitos por câncer de cabeça e pescoço, dois eram de residentes da RGI Rio de Janeiro, mas diferenças de taxas de mortalidade de acordo com a escolaridade foram mais pronunciadas em regiões do interior do estado, em comparação com a região da capital.

Foi encontrado uma relação entre níveis educacionais baixos e taxas de mortalidade mais altas por câncer de cabeça e pescoço, o que sugere que a posição social, medida por esse indicador, tem um impacto negativo nos desfechos de saúde para pessoas afetadas pelo câncer de cabeça e pescoço. Esse achado está em conformidade com outros estudos sobre câncer de cabeça e pescoço e desigualdades, que demonstram que populações menos favorecidas tendem a apresentar maior incidência e maior risco para morte ^5^ ^,^ ^6^.

A maior mortalidade entre os indivíduos com menor escolaridade pode ser parcialmente explicada pela maior incidência de câncer de cabeça e pescoço neste estrato da população e respectivos fatores associados. Estudo brasileiro mostrou que a prevalência do tabagismo, importante fator relacionado à câncer de cabeça e pescoço, foi significativamente maior entre aqueles com escolaridade até 8 anos de estudo (24,2%) quando comparados àqueles com 9 ou mais anos de estudo (15,7%). Além disso, a prevalência de fumantes com consumo intenso de cigarros foi maior entre aqueles com menor escolaridade ^25^. Nossos achados estão em conformidade com Taib et al. ^6^, que propuseram um modelo de desigualdade em saúde adaptado para os câncer de cabeça e pescoço de modo que o contexto social e político das populações e a posição social de um indivíduo levam à exposição de vulnerabilidades diferenciais a fatores de risco, como tabaco e álcool.

A escolaridade também se mostrou um fator determinante na ocorrência de câncer de cabeça e pescoço. O presente estudo corrobora os resultados de estudo caso-controle, realizado no Canadá entre 2004 e 2005 no qual a ocorrência de câncer de cabeça e pescoço foi três vezes maior em indivíduos com menos de 8 anos de escolaridade do que os que concluíram o Ensino Médio ^26^. Ribeiro et al. ^27^ investigaram os fatores preditivos do câncer oral no Brasil entre 2010 e 2013, e verificaram que possuir uma escolaridade de 12 anos ou mais foi fator protetor contra o desenvolvimento do câncer de cavidade oral na população analisada. Dados da *Pesquisa Nacional de Saúde* (PNS) de 2013 mostram que indivíduos com menor nível de escolaridade apresentam menor frequência de higiene bucal adequada, autopercepção negativa da saúde bucal e maior dificuldade para se alimentar devido a problemas dentários ^28^.

No Brasil, persistem desigualdades educacionais associadas à raça, com uma maior proporção de indivíduos pardos e pretos entre os menos escolarizados ^29^. Consequentemente, com base nos resultados de nosso estudo, é esperada uma maior taxa de mortalidade proporcional por câncer de cabeça e pescoço entre pardos e pretos. Com efeito, existem evidências na literatura de que indivíduos negros têm maior chance de serem diagnosticados com câncer de cabeça e pescoço em estágios avançados em comparação aos brancos ^30^ ^,^ ^31^. Além disso, esse grupo também têm uma maior probabilidade de receber cuidados inadequados, enfrentar atrasos no acesso a cuidados de saúde e apresentar mais comorbidades ^9^ ^,^ ^30^. Fakhry et al. ^30^ demonstraram que a raça exerce um impacto diferenciado na morbidade e mortalidade do câncer de cabeça e pescoço. Indivíduos de cor da pele preta apresentaram maior frequência de uso de tabaco e álcool, buscavam o serviço médico em estágios mais avançados da doença e tinham pior sobrevida em comparação com outros grupos raciais. Em São Paulo, verificou-se entre 2003 e 2009 que a mortalidade por câncer de cabeça e pescoço aumentou em mulheres e quase dobrou em indivíduos pretos, ultrapassando a mortalidade em brancos em quase todos os subtipos de tumores de câncer de cabeça e pescoço avaliados ^9^.

Os resultados do presente estudo indicam que os homens apresentam maior risco de mortalidade por câncer de cabeça e pescoço, o que sugere que essa população ainda está mais exposta a fatores de risco, como tabagismo e consumo de álcool e tende a buscar menos serviços de saúde ^28^ ^,^ ^32^. Baseado em uma pesquisa conduzida no Brasil, que avaliou a autopercepção da saúde bucal dos brasileiros, foi observado que homens e indivíduos com baixa escolaridade apresentaram menor frequência de indicadores relacionados à higiene bucal adequada e à percepção de uma boa ou excelente saúde bucal ^28^. Inquérito nacional mostra maior proporção de fumantes entre homens com até 8 anos de estudo, o que parcialmente explica taxas mais elevadas de mortalidade por câncer de cabeça e pescoço nesse grupo ^33^.

Entretanto, estudos apontam que embora a mortalidade entre homens ainda seja alta, é esperado que ocorra aumento expressivo da mortalidade por câncer de cabeça e pescoço entre mulheres nos próximos anos ^9^ ^,^ ^30^ ^,^ ^34^. Isso se deve em parte à mudança no comportamento de estilo de vida e à exposição aos fatores de risco levando em conta o sexo ^33^. Embora a prevalência de tabaco seja menor na população feminina brasileira e tenha estabilizado ao longo dos anos, houve aumento da frequência de consumo abusivo de álcool de 7,8% em 2006 para 11,3% em 2021 ^33^. No Brasil, foi observado entre 2000 e 2013 um crescimento da mortalidade por câncer de cabeça e pescoço entre mulheres de 1,3% ao ano ^34^. Em São Paulo, entre 2003 e 2009 a mortalidade entre mulheres teve crescimento anual de 4,4% ^9^.

Em nosso estudo, foi observado uma redução no efeito protetor da escolaridade na mortalidade câncer de cabeça e pescoço na medida em que a idade avançou. Essa tendência pode ser explicada pelo fato de que, ao passo que as pessoas envelhecem, estão expostas a múltiplos fatores de morbidade além do câncer ^35^. Portanto, espera-se que, com o aumento da idade, as diferenças na mortalidade por câncer de cabeça e pescoço sejam menos influenciadas pelo efeito da escolaridade.

Estudos indicam que o nível de escolaridade tem um impacto significativo no acesso da população aos serviços de saúde básica e especializada ^7^ ^,^ ^32^ ^,^ ^36^. Estudo brasileiro constatou que quanto menor o nível de escolaridade, maior é o risco de acesso precário aos serviços de saúde no Brasil ^7^. Outra pesquisa nacional, com base nos dados da PNS de 2019, descreveu maior prevalência de realizar atividades habituais por motivos de saúde nas duas semanas anteriores à pesquisa (14%) em indivíduos com menor escolaridade. Por outro lado, aqueles com maior escolaridade apresentaram proporções mais altas de consultas odontológicas nos últimos 12 meses (71,9%) e de obtenção de todos os medicamentos prescritos em sua última consulta médica (90%), em comparação com indivíduos sem instrução ou com menor escolaridade ^36^.

As diferenças nas taxas de mortalidade entre menos e mais escolarizados foram maiores em regiões do interior do estado. O Estado do Rio de Janeiro é um dos mais desiguais do país, apresentando um índice de Gini de 0,54 e taxas mais elevadas de analfabetismo concentradas nos municípios das regiões norte, noroeste e serrana ^11^ ^,^ ^37^. Além disso, sabe-se que a assistência à saúde especializada em câncer no estado é concentrada na capital, o que leva os pacientes a se deslocarem para receber o tratamento adequado ^37^. É plausível considerar que pacientes em situações desfavoráveis com residência no interior do estado sejam os mais afetados devido à maior dificuldade de deslocamento em busca de atendimento especializado.

As taxas de mortalidade por câncer de cavidade oral e orofaringe mais altas em ambos os sexos, descritas em nosso estudo, estão em conformidade com dados da literatura. No Brasil, entre 1979 e 2013, foi observado que a mortalidade por esses tumores foi mais alta nas regiões Sul e Sudeste ^34^. No Estado de São Paulo, no período de 2000 a 2018, foi observado um aumento de 34,9% nas disparidades de sobrevida em sítios anatômicos relacionados ao HPV, enquanto houve uma redução de 10,2% e 29,6% no câncer de boca e laringe ^38^.

Limitações a serem consideradas são a qualidade dos dados secundários utilizados para o cálculo das taxas, apesar da boa completude da informação sobre óbitos em comparação a outras bases de dados. É importante mencionar a variação na classificação do câncer de cabeça e pescoço entre estudos, o que pode dificultar a comparação de resultados. Portanto, é necessário levar em conta essas variações na classificação ao comparar estudos sobre o tema para uma interpretação adequada dos resultados ^2^. A técnica de imputação de dados usada no estudo não permitiu a redistribuição dos óbitos, o que pode ter levado a uma subnotificação das taxas de mortalidade estimadas. No entanto, a análise utilizou uma escala mínima de agregação da informação sobre escolaridade, em comparação com estudos que consideram indicadores macrossociais. A imputação da escolaridade reduziu a subestimação das taxas de mortalidade relacionadas a essa variável para o conjunto de tumores analisados.

Outra limitação do estudo diz respeito ao uso das taxas brutas de mortalidade na análise dos dados. A metodologia de imputação empregada na construção dos numeradores das taxas de mortalidade não possibilita o uso de uma população padrão para ajustes. De qualquer forma, ressalta-se que as razões das taxas de mortalidade por escolaridade, foco do presente estudo, foram ajustadas por sexo e idade, ou seja, são estimativas geradas considerando as variações nessas características demográficas.

Em conclusão, foi constatado que a mortalidade por câncer de cabeça e pescoço no Estado do Rio de Janeiro está correlacionada com menor nível de escolaridade, especialmente entre os homens. Medidas de prevenção primária, com a promoção de uma alimentação saudável, o abandono do tabagismo e redução do consumo de álcool, devem ser implementadas como estratégias focadas em homens com baixa escolaridade para reduzir o impacto da mortalidade por câncer de cabeça e pescoço no Estado do Rio de Janeiro.
